# Structured physiotherapy including a work place intervention for patients with neck and/or back pain in primary care: an economic evaluation

**DOI:** 10.1007/s10198-018-1003-1

**Published:** 2018-08-31

**Authors:** Sanjib Saha, Birgitta Grahn, Ulf-G. Gerdtham, Kjerstin Stigmar, Sara Holmberg, Johan Jarl

**Affiliations:** 1 Health Economics Unit, Department of Clinical Science (Malmö), Lund University, Medicon Village, Scheelevägen 2, 22381 Lund, Sweden; 2 Department of Clinical Sciences Lund, Orthopedics, Lund University, Lund, Sweden; 3 Department of Research and Development, Region Kronoberg, Växjo, Sweden; 4 Centre for Economic Demography, Lund University, Lund, Sweden; 5 Department of Economics, Lund University, Lund, Sweden; 6 Department of Health Sciences, Physiotherapy, Lund University, Lund, Sweden; 7 Skåne University Hospital, Lund, Sweden; 8 Division of Occupational and Environmental Medicine, Institute of Laboratory Medicine, Lund University, Lund, Sweden

**Keywords:** Cost–effectiveness analysis, Cost–utility analysis, Return-to-work, Musculoskeletal pain, Quality-adjusted life-years, H43, I10, I18

## Abstract

A cluster-randomized controlled trial, WorkUp, was conducted for working-aged patients at risk of sick leave or on short-term sick leave due to acute/subacute neck and/or back pain in Sweden. The purpose of WorkUp was to facilitate participants to stay at work or in case of sick leave, return-to-work. The aim of this study was to study whether the WorkUp trial was cost-effective. Patients in the intervention and reference group received structured evidence-based physiotherapy, while patients in the intervention group also received a work place dialogue with the employer as an add-on. The participants, 352 in total, were recruited from 20 physiotherapeutic units in primary healthcare in southern Sweden. The economic evaluation was performed both from a healthcare and a societal perspective with a 12-month time frame with extensive univariate sensitivity analyses. Results were presented as incremental cost–effectiveness ratios (ICER) with outcomes measured as quality-adjusted life-years (QALY) and proportion working for at least 4 weeks in a row without reported sick leave at 12-month follow-up. From the healthcare perspective, the ICER was €23,606 (2013 price year) per QALY gain. From the societal perspective the intervention was dominating, i.e.. less costly and more effective than reference care. Bootstrap analysis showed that the probability of the intervention to be cost-effective at €50,000 willingness-to-pay per QALY was 85% from the societal perspective. Structured evidence-based physiotherapeutic care together with workplace dialogue is a cost-effective alternative from both a societal and a healthcare perspective for acute/subacute neck and/or back pain patients.

*Trial registration* ClinicalTrials.gov: NCT02609750.

**Electronic supplementary material:**

The online version of this article (10.1007/s10198-018-1003-1) contains supplementary material, which is available to authorized users.

## Introduction

Musculoskeletal pain (MSP) is one of the most common causes of sick leave in the Western world. Approximately 20% of the Swedish population suffers from neck and/or back pain [1, 2]. The total cost of musculoskeletal disorders in Sweden was estimated to 102 billion SEK in 2012 where sick leave constituted two-thirds of the total costs [3].

It is therefore important to identify how individuals with MSP can maintain or regain ability to work as well as Return-To-Work (RTW) from sick leave. The WorkUp trial (ClinicalTrials.gov: NCT02609750), was conducted in southern Sweden during 2013–2015. It was a cluster Randomized Controlled Trial (RCT) targeting working-aged patients with acute/subacute neck and/or back pain and at risk for sick leave or on shorter sick leave. Using structured physiotherapeutic interventions and a workplace intervention termed “Convergence Dialogue Meetings” (CDM) which integrated an early dialogue with the employer to identify the needs for workplace adjustments, the intervention aimed to maintain work ability or, if sick-listed, facilitate RTW. At 12-month follow-up, more patients in the intervention group were able to work at least 4 weeks in a row compared to the reference group [4].

This raises the question whether the intervention, besides being more effective, also is a better use of society’s available scarce resources compared to reference care, i.e.. whether it is cost-effective. Published evidence is inconclusive on the cost–effectiveness of different RTW interventions for MSP patients [5, 6]. For example, multi-stage RTW programs were shown to be effective and potentially cost-effective compared to reference care for individuals with low-back pain in the Netherlands [7] and in Canada [8], while similar RTW programs were found to be expensive and not cost-effective compared to reference care in Denmark [9] and in the Netherlands [10].

The objective of the current study was to perform an economic evaluation of the WorkUP trial from both a societal and a healthcare perspective with a 12-month time frame.

## Materials and methods

### The WorkUp trial

The trial was conducted during 2013–2015 in the southern part of Sweden [4]. A total of 32 Primary HealthCare (PHC) centers linked to 20 physiotherapeutic units took part in the trial. WorkUp had a two-armed, pair-wise cluster-randomized design. All PHC centers were categorized based on size, number of registered patients and the socioeconomic standard (CNI—Care Need Index) and healthcare need (ACG—Adjusted Clinical Groups) of the covered population and, based on this, grouped in pairs. A randomization in each pair was made into the two arms in the study (intervention and reference) leaving ten physiotherapy units in each arm. Power analyses were performed based on the primary outcome work ability, where sick leave was estimated to be decreased by 30% in the intervention group and 10% in the reference group. This resulted in that 20 units and 500 included patients were required (80% power, *p* < 0.05). Inclusion criteria for the patients were (1) applying for physiotherapy due to acute/subacute symptoms (< 12 weeks) in the neck and/or back; (2) working at least 4 weeks during the last year; (3) not on sick leave or sick-listed for no more than 60 consecutive days; (4) at risk for developing long-standing disability measured by the Örebro Musculoskeletal Pain Screening Questionnaire (scoring ≥ 40) and (5) able to communicate in Swedish. The exclusion criteria were known abuse, other acute illness, and pregnancy. The total number of included patients at baseline was 352 comprising 146 in the intervention group and 206 in the reference group. At 12-month follow-up, there were 132 patients (90%) in the intervention group and 183 (89%) in the reference group. Detailed characteristics of the participants at baseline and 12 months are presented in Table 1.

**Table 1:** Characteristics of the participants in the WorkUp trial at baseline and 12 months follow-up

	Baseline								Follow-up (12 months)							
	Intervention (*N* = 146)				Reference (*N* = 206)				Intervention (*N* = 137)				Reference (*N* = 184)			
	Mean	Sd	*N*	%	Mean	Sd	*N*	%	Mean	Sd	*N*	%	Mean	Sd	*N*	%
Age	43.8	11.7			43.7	12.6			43.85	11.45			44.02	12.28		
QALY (Swedish tariff)	0.771	0.12			0.760	0.12			0.879	0.09			0.847	0.12		
QALY (UK tariff)	0.526	0.29			0.490	0.30			0.742	0.20			0.691	0.26		
Sex																
Male			54	37			68	33			53	39			62	34
Female			92	63			138	67			84	61			121	66
Civil status																
Single			34	23			47	23			32	23			43	23
Married/living together			112	77			157	76			105	77			139	76
Born in Sweden																
Yes			132	90			173	84			124	90			158	86
No			14	10			32	16			13	10			25	14
Education																
Secondary			16	11			14	7			15	11			12	6
Upper secondary			69	47			107	52			34	47			97	53
College/university			28	19			49	24			26	19			43	23
Others			33	23			35	17			32	23			31	27
Diagnosis																
Neck and shoulder			27	18			49	24			27	20			44	24
Neck and lower back			9	6			12	6			6	4			11	6
Low-back ischia			102	70			140	68			96	70			124	67
Generalized muscle			8	5			5	2			8	6			5	3
Have job																
Yes			142	97			194	94			133	97			173	94
No			4	3			11	6			4	3			10	6
Sick leave																
No			93	65			131	64			100	87			138	83
Yes			51	35			74	36			15	13			29	17

*N* number; Sd standard deviation; % percentage

Patients in the intervention and reference group received structured evidence-based treatment by a physiotherapist based on biopsychosocial perspective. The intervention group received a workplace intervention: “Convergence Dialogue Meeting (CDM)” in addition [11]. CDM is a dialogue in three steps between the patient, the physiotherapist, and a representative from the workplace. The patients were first interviewed by the physiotherapist to identify factors that can affect their work ability. Thereafter, an interview with the patient’s supervisor or manager was conducted by the physiotherapist to identify possible work place adjustments that could facilitate remaining at work or RTW. In the third step, a meeting was initiated with the patient, the supervisor and the physiotherapist to discuss which work place adjustments were needed to support work ability and to agree on a plan for this.

All participants were followed during 1 year and self-reported data were collected at baseline, 3, 6 and 12 months including self-rated health and number of healthcare visits since the last follow-up. Sick leaves were collected on a weekly basis using short text messages. The patients reported their current work ability by answering how many days they were on sick leave that particular week. At 3, 6 and 12 months, clinical examination by a physiotherapist was also conducted.

### Outcome measures

We used quality-adjusted life-years (QALY) measured by the EQ-5D questionnaire at baseline and 12-month follow-up as the main outcome for this study in the cost–utility analysis (CUA). Given the randomization of the patients into the different treatments and that there is no significant difference in QALY at baseline; we measure the effect on Health-Related Quality of Life (HRQoL) as the difference between groups at follow-up.

The EQ-5D is a generic preferences-based measure of HRQoL which comprise five attributes: mobility, self-care, usual activities, pain/discomfort and anxiety/depression. Each attribute has three levels: no problems, some problems, and extreme problems, thus defining 243 possible health states. These health states are translated into a score varying between 0 (equivalent of dead) and 1 (perfect health) using the recently developed experience-based Swedish tariff [12]. Alternative tariffs, such as the often-used UK tariff [13], is hypothetical and thereby give different results [14]. To facilitate comparison with other studies the results are also shown using the UK tariff.

We also use the self-reports by short text messages on work ability at 12-month follow-up as a secondary outcome to be in line with the effectiveness study [4] when conducting the cost–effectiveness analysis (CEA). This outcome variable is defined as having worked or been available to the labor market for at least 4 weeks in a row at 12 months follow-up without any reported sick leave [15]; a yes or no dichotomous response.

### Costs and service use

The economic evaluation was conducted both from a healthcare and a societal perspective. In the societal perspective, all costs are included irrespective of who is burdened by them, while the healthcare perspective is only concerned with costs burdening the healthcare sector (although the health benefits of the patients are the effectiveness measure) [16].

Healthcare utilization outside the intervention during the first year after treatment start was assessed based on patient self-reported information (questionnaires at 3-, 6-, and 12-month follow-up). The questionnaires included number of healthcare visits separately for physicians, nurses, physiotherapists, psychologists, occupational therapists and behavioral therapists. Costs were calculated by multiplying the respective number of visits by standard costs according to the Swedish Association of Local Authorities and Regions database [17]. For visits where costs for healthcare personnel were not available, costs according to professional organizations were used. The cost of CDM is the only intervention cost.

Production loss is incurred as individuals are unable to perform their regular work due to sickness, measured in the study by weekly self-reported sick leave. Production loss was valued according to gross salary (including taxes) for the individual on sick leave, an approach termed the human capital approach [18]. Since patient’s income was not available from the data, estimation of production loss was based on the average salary in Sweden. The cost per hour of lost working time was estimated at €23 based on an average pre-tax salary of €3538 (30,600 SEK) per month according to Swedish National Mediation Office [19]. Participants’ time and travel costs as well as cost of pharmaceuticals and alternative medications were not considered due to lack of data. Therefore, patient’s perspective was not considered while conducting this economic evaluation. All costs were estimated in Swedish Kronor (SEK) in 2013 price year and converted to Euros (EUR) using an 8.65 SEK/EUR exchange rate [20]. Neither costs nor the outcomes were discounted as the time frame of the study was 1 year.

### Statistical analyses

Incremental cost–effectiveness ratios (ICER) were calculated as the ratio of the incremental costs and incremental effects. The ICER represents the additional costs needed to gain one extra unit of effect in the intervention group compared to the reference group. Uncertainty surrounding the incremental costs and effects were estimated using non-parametric bootstrapping with 5000 replications [21]. The 95% confidence intervals (95% CI) around the mean cost differences were estimated using the Approximate Bootstrap Confidence (ABC) algorithm [22] followed by Student’s t test. Bootstrapped incremental cost–effect pairs were plotted on a cost–effectiveness plane (CE plane), which shows the difference in effect on the horizontal axis and the difference in costs on the vertical axis [23] of the two interventions. If all points are in the southeast or the northwest quadrant the choice between the interventions is clear. In the southeast quadrant, the intervention is both more effective and less costly than reference care and the intervention is considered to dominate reference care, while the opposite is true in the northwest quadrant. In the northeast and southwest quadrants, the choice depends on the valuation of the outcome which is defined as what society is willing to pay to gain one unit of effect [16, 24]. Cost–effectiveness acceptability curve (CEAC) was also estimated which shows the probability that the intervention is cost-effective in comparison with reference care for a range of Willingness-To-Pay (WTP) thresholds. The economic evaluation has been conducted following the Consolidated Health Economic Evaluation Reporting Standards (CHEERS) statement [25]. The statistical analyses were conducted in STATA 14 (StataCorp, Collage Station, TX, USA).

### Sensitivity and subgroup analyses

We performed several sensitivity and subgroup analyses to capture uncertainties around the findings. The sensitivity and subgroup analyses were based on the main cost–utility analysis.

- *Using UK tariff for QALY estimation* The UK tariff [13] is the most used tariff in the scientific literature and based on the time-trade-off approach with hypothetical questions. We perform this analysis in order to increase comparison of results with other studies (analysis 1).
- *Controlling for baseline QALY* The baseline QALYs were numerically but not statistically different between the intervention and reference group (Table 1). In the base case estimate, we treat this as being equal in size while in this sensitivity analysis we control for the numerical difference (analyses 2a, b).
- *Patients with sick leave* Results are reported separately for the participants who were/were not on sick leave at baseline (analyses 3a, b).
- *Excluding outliers—cost* 5% of the participants in both the intervention and the reference group with the highest cost of healthcare utilization are excluded as well as all participants with zero healthcare utilization cost (lowest cost) (analyses 4a, b).
- *Excluding outliers—QALY* The highest and lowest 2.5% in terms of QALY gain and loss are excluded (analysis 5).
- *Subgroup analyses—gender* Results are reported separately for men and women (analyses 6a, b) as healthcare seeking behaviors differ according to gender.
- *Subgroup analyses—age* Results are reported separately for participants lower than 41 years old and participants higher/equal 41 years old (Sweden’s life expectancy at birth is 82 years [26]) (analyses 7a, b) as health problems are expected to rise with increasing age.

## Results

### Costs and effects

In terms of healthcare utilization, some non-significant differences between the groups can be noted (Table 2). The mean costs for physician and physiotherapists visits were higher for the intervention group, whereas the reference group had more psychologists visits, translating into higher costs (unit costs and sources are presented in Table S1 in the supplementary material). The cost of CDM comprises time for a physiotherapist with 1 h of preparation time per patient. The intervention group had higher total healthcare cost than the reference group (*p* = 0.02). In terms of productivity loss, this was higher in the reference group compared to the intervention group although not statistically significant.

**Table 2:** Mean cost, effect and differences by bootstrap (5000) for intervention and reference group

	Intervention group		Reference group		Difference	
	Mean	SE	Mean	SE	Mean	95% CI
Cost						
Healthcare perspective						
General practitioner	644.07	267	255.77	57	388	− 151 to 927
Medical specialist	145.26	59	65.89	32	79	− 52 to 211
Psychologists	62.61	46	106.89	51	− 44	− 178 to 90
Physiotherapists	326.01	72	293.00	65	33	− 157 to 223
Other healthcare professionals	90.17	31	64.89	21	37	− 47 to 98
CDM cost	312.56	7.56	0		313	
Total cost (healthcare perspective)	1566	308	786.45	125	779	123 to 1435^∝^
Societal perspective						
Productivity loss	10,624	1097	11,684	944	− 1,060	− 3906 to 1798
Total cost (societal perspective)	12,190	1134	12,470	961	− 280	− 3212 to 2653
Effect						
Work continuously last 4 weeks	86%		74%		12%^δ^	3 to 20%
QALY (Swedish tariff)	0.879	0.008	0.847	0.009	0.033	0.006 to 0.058^∂^
QALY (UK tariff)	0.742	0.018	0.691	0.020	0.052	− 0.005 to 0.11

*SE* standard error of mean

^∝^*p* = 0.02; ^δ^*p* = 0.01 (proportion test); ^∂^*p* = 0.009

There was a significant higher increase in QALY after 12 months in the intervention group compared to the reference group (0.033, *p* = 0.01) using the Swedish tariff (Table 2). 86% of the participants in the intervention group scored positively on the work ability outcome “working for at least 4 weeks in a row at 12-month follow-up without reported sick leave” compared to 74% of the reference group (*p* = 0.01).

### Cost–utility analysis

**Fig. 1:**
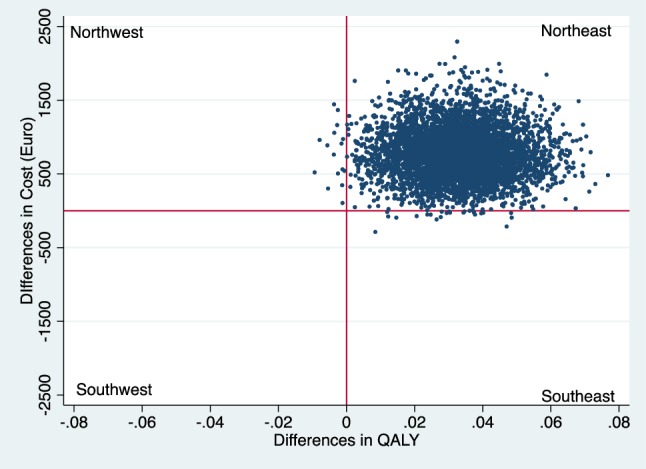
CE-plane from healthcare perspective (northeast 99% and southeast 1%)

**Fig. 2:**
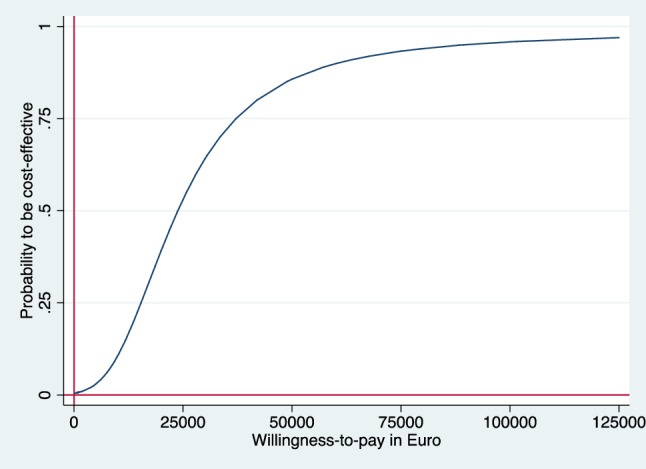
CEAC form healthcare perspective. CEAC indicating the probability of the intervention being cost-effective at different values (€) of willingness-to-pay per QALY gain

There was a significant difference in QALY at 12 months in favor of the intervention (Table 3), and the cost difference of €779 resulted in an ICER of €23,606 per QALY from the healthcare perspective. The CE plane shows that almost all incremental CE-pairs (bootstrapped) are located in the northeast quadrant (99%) indicating that the intervention was more costly and more effective (Fig. 1). The CEAC shows that given a WTP of €50,000 for a QALY, the intervention has almost 80% probability of being cost-effective from the healthcare perspective (Fig. 2).

**Table 3:** Differences in pooled mean costs, effects (95% CI), and incremental cost–effect ratios (ICERs)

Perspective	Effectiveness measures	Cost difference		Effect difference		ICERs
		∆C	95% CI	∆E	95% CI	
Healthcare	Work continuously last 4 weeks	779	123 to 1435	12%	3 to 20%	65
	QALY (Swedish tariff)	779	123 to 1435	0.033	0.006 to 0.058	23,606
Societal	Work continuously last 4 weeks	− 280	− 3212 to 2653	12%	3 to 20%	Dominant
	QALY (Swedish tariff)	− 280	− 3212 to 2653	0.033	0.006 to 0.058	Dominant

**Fig. 3:**
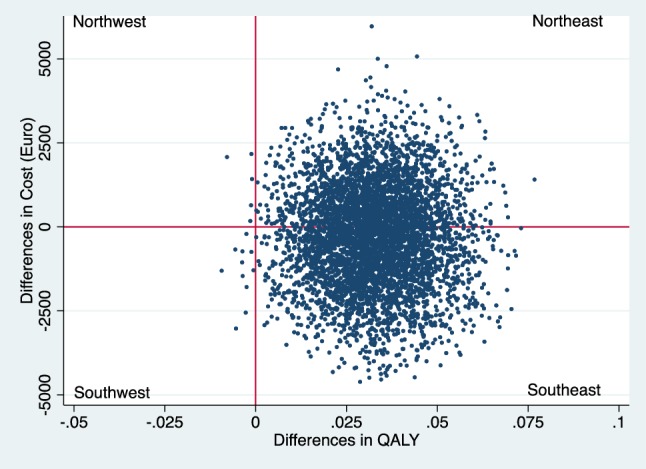
CE-plane from societal perspective (northeast 43%, southeast 56% southwest 0.2%, northwest 0.1%)

**Fig. 4:**
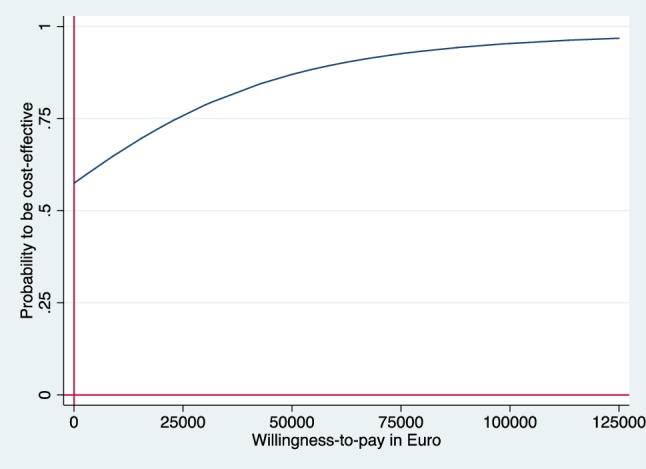
CEAC form societal perspective. CEAC indicating the probability of the intervention being cost-effective at different values (€) of willingness to pay per QALY gain

From a societal perspective, the intervention was the dominant option, meaning the intervention was more effective compared to reference care but not more costly. The incremental CE-pairs were mostly located in the southeast quadrant (56%) and northeast quadrant (42%) of the CE-plane (Fig. 3). The CEAC shows that at €50,000 WTP for a QALY the intervention has almost 85% probability of being cost-effective from a societal perspective (Fig. 4).

### Cost–effectiveness analysis

The CEA from the healthcare perspective showed an ICER of €65 for RTW, meaning that an additional 65 euro was needed to increase the individual’s likelihood of working for at least 4 weeks in a row at 12-month follow-up without reported sick leave by 1% point, compared to reference care. From the societal perspective, the intervention dominated reference care meaning that the intervention was less costly (not statistically significant) and more effective.

### Sensitivity and subgroup analyses

The results of the sensitivity and subgroup analyses are presented in Table 4. The sensitivity analyses were based on the CUA. In the subgroup analyses from the healthcare perspective, the healthcare cost does not differ between the two groups potentially due to small sample size. A substantial gender difference can be noted where the intervention is very cost-effective for men but not at all for women.

**Table 4:** Sensitivity analyses from both healthcare and societal perspective in incremental cost–effect ratios (ICERs)

Analysis no.	Scenarios	Sample size^a^		Changes in effect (QALY gain)	Healthcare		Societal	
		Intervention group	Reference group		Changes in cost	ICER	Changes in cost	ICER
	Base case	115/146	172/206	0.033 (0.01 to 0.06)	779 (123 to 1435)	23,606	− 280 (− 3212 to 2653)	Dominant
1	Using the UK tariff for QALY calculation	115/146	172/206	0.052 (− 0.01 to 0.11)	779 (123 to 1435)	14,981	− 280 (− 3212 to 2653)	Dominant
2a	Using the UK tariff and controlling for differences at baseline	114/146	171/206	0.02 (− 0.05 to 0.09)	779 (123 to 1435)	38,950	− 280 (− 3212 to 2653)	Dominant
2b	Using the Swedish tariff and controlling for differences at baseline	114/146	171/206	0.02 (− 0.01 to 0.05)	779 (123 to 1435)	38,950	− 280 (− 3212 to 2653)	Dominant
3a	Only patients on sick leave at baseline	42/51	60/74	0.025 (− 0.02 to 0.07)	687 (− 282 to 1657)	27,480	1196 (− 4210 to 6604)	47,840
3b	Only patients not on sick leave at baseline	72/93	111/113	0.04 (0.01 to 0.07)	439 (− 100 to 1643)	10,975	− 1407 (− 4870 to 2055)	Dominant
4a	Removing outliers in both groups (highest 5% healthcare cost)	104/135	165/199	0.04 (0.02 to 0.06)	296 (47 to 545)	7400	− 779 (− 3727 to 2169)	Dominant
4b	Removing outliers in both groups (no healthcare cost)	63/94	109/143	0.04 (0.01 to 0.07)	292 (267 to 316)	7300	183 (− 3294 to 3660)	4575
5	Removing outliers in both groups in QALY gain (highest and lowest 2.5%)	108/108	167/167	0.013 (− 0.01 to 0.04)	952 (99 to 1806)	73,231	− 758 (− 4162 to 2645)	Dominant
6a	Men only	38/54	58/68	0.056 (0.02 to 0.09)	4 (− 598 to 607)	71	− 2632 (− 7444 to 2179)	Dominant
6b	Women only	77/92	114/138	0.02 (− 0.01 to 0.05)	1234 (255 to 2213)	61,700	1062 (− 2691 to 4817)	53,100
7a	Age lower than 41	40/58	71/88	0.03 (− 0.02 to 0.07)	1032 (− 354 to 2420)	34,400	1605 (− 3402 to 6614)	53,500
7b	Age 41 and higher	75/88	101/118	0.04 (0.01 to 0.07)	600 (− 18 to 1218)	15,000	− 1531 (− 5157 to 2095)	Dominant

^a^Same sizes are presented for number of participants available for QALYs first, followed by number of participants available for cost estimation

From the societal perspective, the results were sensitive for the participants who were on sick leave at the baseline and zero healthcare utilization cost. For women and participants younger than 41 years of age, the findings were also sensitive. The intervention is no longer dominant in these scenarios although still cost-effective by most standards (see “Discussion”).

## Discussion

We performed an economic evaluation of the WorkUp trial focusing on health-related quality of life and work ability for participants with acute/subacute neck and/or back pain from both a healthcare and a societal perspective. The aim of the WorkUp trial was to study whether a work place intervention could impact work ability. Patients were recruited in an early stage of disability, i.e.. not being on sick leave or only having a short period of sick leave. Around 65% of patients in both the intervention and reference group were not on sick leave at baseline. Thus, work ability is a more adequate main outcome for this study than RTW.

We choose to perform both a CUA and CEA because CEA which uses a natural unit as outcome measure is more relevant to clinicians [27] while CUA is more relevant to decision-makers [28] as this enables comparison between different interventions. Since the main purpose of economic evaluation, in general, is to facilitate decision-makers to take informed decision, we prefer CUA over CEA and thus the uncertainties around the base case findings were explored for CUA only by CE planes, CEAC curves and sensitivity and subgroup analyses.

The intervention had high potential to be cost saving from the societal perspective irrespective of which effectiveness measure was used, whereas mixed results were found from a healthcare perspective. From the healthcare perspective, if we consider the outcome as “working for at least 4 weeks in a row at 12-month follow-up without reported sick leave” the ICER was only €65. The ICER was €23,606 when considering the effectiveness as QALY, meaning that €23,606 is required to gain an additional QALY in the intervention group compared to the reference group. Cost–effectiveness is determined based on the value of the effectiveness measure, also known as the cost–effectiveness threshold. Sweden does not have a formal threshold for cost–effectiveness ratios although the Swedish National Board of Health and Welfare have considered interventions costing less than 500,000 SEK (€57,803) per QALY gained as cost-effective [29]. The World Health Organization regards an ICER lower than the gross domestic product per capita (€34,394 in Sweden in 2013) as very cost-effective [30]. Therefore, the WorkUp intervention can be considered as cost-effective from the healthcare perspective as well.

There are some economic evaluations of participatory RTW interventions for employees with musculoskeletal disorders [7, 8, 31, 32]. Steenstra et al. found that a workplace intervention was cost-effective for patients with low-back pain in terms of work-related measures like RTW compared to reference care, but no significant effect was found in QALY [7]. This is in line with our findings using the UK tariff although Steenstra et al. used the Dutch tariff [7]. Aligned with other studies we found that the healthcare cost is significantly higher in the intervention group than the reference group [9, 32], normally due to the cost of the intervention as such. It is expected that the 1-h preparation time for conducting the CDM, will be significantly reduced when the physiotherapist becomes more experienced using the CDM method. The economic benefit from the productivity gain, however, was greater than the intervention costs and thus a favorable ICER was obtained from societal perspective in this study. This is in line with one Danish study which showed that productivity gain was higher than the intervention cost in a coordinated and tailored rehabilitation program for workers on sick leave due to MSP [31].

The sensitivity analysis showed that the intervention was not cost-effective for women from a healthcare perspective (analysis 6b). It is known that gender differences exist in health-seeking behaviors and women visit healthcare personnel to a greater extent than men [33]. The results are sensitive to removing the outliers in healthcare utilization. The phenomenon of a few cases driving the costs is common in epidemiological studies of occupational back pain and has also been noted in economic evaluations. An intervention to prevent low-back pain [8] where some high-cost participants drew the costs causing the intervention not to be cost-effective. We also found that for younger participants, the intervention was not dominant in the societal perspective. Back and neck pain are more common in younger populations and back pain falls with age [34]. This could be the reason for low QALY gains in the younger participants and higher societal cost. It should be noted that the trial was not powered to detect differences between subgroups in terms of costs and effects and any loss of significance in the subgroup analyses, compared to the base case estimations, might be due to this. These results should therefore be interpreted with caution.

We found that using the Swedish tariff provided significant QALY difference between the intervention group and the reference group at 12-month follow-up, but not when using the UK tariff. It is difficult to explain the different results between tariffs. One possible explanation could be that the hypothetical UK tariff provides more negative values for same attributes than the experience-based Swedish tariff. Therefore, the mean QALY value is lower in the UK tariff (Table 2), but also lower for most specific health states [14]. The Swedish tariff has been shown to be more accurate than the UK tariff in the Swedish populations [35] and is therefore considered the most appropriate tariff in the current study.

To the authors’ knowledge, this is the first economic evaluation of a structured physiotherapy intervention in PHC combined with work place intervention CDM for participants with acute/subacute neck and/or back problem in Sweden. An important strength of the current study is that the economic evaluation was conducted alongside the RCT, therefore limiting the risk of bias. Another strength is that this was a pragmatic study, resembling the actual healthcare practice in PHC for participants with health problems. We used two different tariffs (the UK and Swedish) for the estimation of QALY which could be seen as a positive aspect of the study.

The study also has some limitations. The study showed no significant effect in terms of costs, QALY gain when using the UK tariff, or the Swedish tariff when controlling for the (insignificant) difference at baseline (sensitivity analyses 1 and 2b). Therefore, cautious interpretation is required of the actual effect of the intervention in terms of QALY. The RCT was underpowered to detect cost and QALY differences because the power calculation was based on work- and health-related outcomes. Cost data usually follow a highly skewed distribution, implying a need for larger sample sizes in cost–effectiveness studies as compared to effectiveness studies [36]. Moreover, the correlation of the difference in cost and difference in effects between two groups as well as maximum willingness to pay are important aspects to consider in power calculations for economic evaluations [37, 38]. These issues were not considered in the WorkUp trial and the power calculation was based solely on work- and health-related outcomes. However, as a significant treatment effect was found even though the RCT did not reach the participation rate needed and that the current study has the objective to assess the likelihood that the intervention is cost-effective and not to test a particular hypothesis regarding cost–effectiveness [39], the sample size of the clinical trial is considered acceptable. As mentioned above however, this issue is aggravated in the subgroup analyses. Other limitations include lack of some costs such as medication use, time and travel costs and difficulty of generalizing the results of this study to other contexts. The intervention was specifically tailored for the study population and the trial was performed in PHC in the Swedish context. When using the combined intervention (physiotherapy and CDM) in a different setting, the population characteristics as well as social, political, cultural and healthcare services in which the program will be implemented and used need to be taken into consideration.

The follow-up duration of the trial was 1 year which was deemed appropriate to capture the short-term effect of the intervention. However, it may be too short to observe the long-term effect of the intervention. In a future study, it would be interesting to know whether the intervention still remains cost-effective or not with long-term follow-up data. The self-reported information on healthcare utilization may have been affected by recall bias even though the information was collected in every 3 months. A register-based study can provide actual estimates of the patients’ healthcare utilization and thus can validate the self-reported information.

RCTs are increasingly common in PHC research to study measures to maintain work ability for employees at risk of sick leave and facilitate RTW for employees on sick leave. But it is important to also assess costs and cost–effectiveness apart from work- and health-related outcomes of these trials. Moreover, the results of economic evaluations are essential to influence policymakers for implementation of new interventions on how work ability can be supported, also at an early stage. The intervention provided in the WorkUp trial reduced sick leave at 12-month follow-up and was found economically beneficial from both a healthcare and a societal perspective. Hence, implementation of this intervention may potentially reduce societal costs while improving patient quality of life, a win–win scenario. Uncertainties in results and long-term effects, however, warrant further studies in the Swedish setting before decision on broad implementation can be made.

## Electronic supplementary material

Below is the link to the electronic supplementary material.

Supplementary material 1 (DOCX 52 KB)
