# Supplementary material for: Structured physiotherapy including a work place intervention for patients with neck and/or back pain in primary care: an economic evaluation
Source: Eur J Health Econ. 2018 Aug 31;20(2):317–27. doi: 10.1007/s10198-018-1003-1 (PMC6438933; doi:10.1007/s10198-018-1003-1)
Supplement: Supplementary file 1 — Supplementary material 1 (DOCX 52 KB) [file 10198_2018_1003_MOESM1_ESM.docx]

**Supplementary materials**

Table S1: Unit costs for different healthcare professionals

|  | Swedish Krona (2013) | Euro (2013) | Source |
| --- | --- | --- | --- |
| General physician | 3,165 | 366 | 1 |
| Specialist physician | 3,669 | 424 | 1 |
| Psychologists | 3,594 | 415 | 1 |
| Physiotherapist | 876 | 101 | 1 |
| Others (occupational therapists, chiropractor, homeopath, acupuncture, dietician) | 876 | 101 | 1 |

Source:

1. <https://statva.skl.se/KPP_somatik_publik.html>
